# Heat Source Forecast of Ball Screw Drive System Under Actual Working Conditions Based on On-Line Measurement of Temperature Sensors

**DOI:** 10.3390/s19214694

**Published:** 2019-10-29

**Authors:** Zhenjun Li, Zechen Lu, Chunyu Zhao, Fangchen Liu, Ye Chen

**Affiliations:** 1 School of Mechanical & Automation, Northeastern University, Shenyang 110819, China; lizhenjungongzuo@163.com (Z.L.); 1610091@stu.neu.edu.cn (Z.L.); 1870156@stu.neu.edu.cn (F.L.); 2 School of Mechanical Engineering and Automation, Liaoning University of Technology, Jinzhou 121001, China; cymaxim@163.com

**Keywords:** ball screw drive system, dynamic thermal network model, inverse method, optimizing prediction analysis, temperature sensor

## Abstract

In view of the time-varying complexity of the heat source for the ball screw feed system, this paper proposes an adaptive inverse problem-solving method to estimate the time-varying heat source and temperature field of the feed system under working conditions. The feed system includes multiple heat sources, and the rapid change of the moving heat source increases the difficulty of its identification. This paper attempts to develop a numerical calculation method for identifying the heat source by combining the experiment with the optimization algorithm. Firstly, based on the theory of heat transfer, a new dynamic thermal network model was proposed. The temperature data signal and the position signal of the moving nut captured by the sensors are used as input to optimize the solution of the time-varying heat source. Then, based on the data obtained from the experiment, finite element software parametric programming was used to optimize the estimate of the heat source, and the results of the two heat source prediction methods are compared and verified. The other measured temperature points obtained by the experiment were used to compare and verify the inverse method of this numerical calculation, which illustrates the reliability and advantages of the dynamic thermal network combined with the genetic algorithm for the inverse method. The method based on the on-line monitoring of temperature sensors proposed in this paper has a strong application value for heat source and temperature field estimation of complex mechanical structures.

## 1. Introduction

As a key component of precise transmission and positioning, the feed system of the Computerized Numerical Control (CNC) machine tool plays a vital role in the positioning accuracy of the machine tool. It reduces the non-cutting operation time and tool replacement time and makes the processing more convenient [1]. A large number of research scholars have devoted themselves to improving the machining accuracy of machine tools. It has been found that the accuracy of machine tools decreases due to temperature rise, and the errors caused by thermal errors account for more than 40% of the total errors [2,3]. The main reason for the thermal error is the friction heat generation of the bearing and the screw nut in the feed system. Under high-speed working conditions, the preload and stiffness of the ball screw feed system change nonlinearly under the influence of heat, which seriously affects the positioning accuracy and working life of the machine tool [4,5,6]. The mechanical and thermal characteristics of the feed system are interrelated, so it is increasingly important to predict the dynamic characteristics of heat source and temperature field under working conditions.

To analyze the temperature field and thermal deformation of the machine tool, it is necessary to determine the heat source of the feed system. The ball screw feed system consists of two main components: a fixed heat source (bearing) and moving heat source (screw nut). Bearings are the key component of feed system of high-speed CNC machine tool. The temperature variation of bearing has a great impact on the performance of bearing system, and affects many key parameters of bearing [7,8,9,10]. Palmgren [11] had made a great contribution to the calculation of bearing temperature. The empirical formula for calculating friction moment was fitted through experimental analysis. Hernot et al. [12] discovered a new method for calculating the heat generation rate of bearings. The influence of the rotational moment and gyroscopic moment was taken into account in this calculation method, and the transient thermal behavior of bearings is calculated more accurately. On the basis of the Jones’ original model, Harris [13] established the quasi-static equilibrium equation of high-speed ball bearings, considering not only the influence of the inertial force but also the high-speed state and Hertz contact deformation. Jin et al. [14] develop a general thermal characteristic evaluation method. The thermal contact resistance between the balls and the inner and outer rings of the supporting bearing is calculated using the Hertzian theory and JHM method. A lot of the above research work is helpful for determining the heat generation rate of the rolling bearing of heat source, but does not consider the influence of the thermal effect on the heat source performance, and that the heat friction heat of the heat source changes nonlinearly with time.

In recent years, numerical analysis methods, such as the finite element method, finite difference method, and thermal network etc. have been widely used to analyze the structural temperature field [15,16,17,18,19,20]. Uhlmann [21] proposed a new thermodynamic model, which used finite element simulation to calculate the thermodynamic behavior of the high-speed cutting center. Zheng [22] established an optimized thermal resistance network model to estimate the temperature field of the bearing system. The optimization model was verified, and the bearing temperature rise can be well predicted. Liu [23] established the thermal network model of the spindle bearing model to calculate the temperature distribution. The thermal structure coupling effect of transient thermal analysis is considered. The experimental results show that the temperature calculated by the transient thermal network model is more accurate than that calculated by the steady-state model. The above calculation method calculates the heat generation of the heat source according to the empirical formula. The heat generation of the heat source varies nonlinearly under the actual working condition, so it can not reflect the temperature field under the actual working condition. Therefore, it is particularly important to determine the heat source of the feed system.

For engineering problems, the inverse method is becoming more and more common to solve scientific and engineering problems, which has attracted the wide attention of scholars at home and abroad. The inverse method is used to estimate the heat source, temperature field, and thermal boundary conditions of engineering problems, and it is not easy to measure or calculate the parameters that are not accurate enough [24,25,26]. Huang et al. [27] used the experimental data to solve the three-dimensional heat conduction problem by using the inverse method based on the simulation and measurement temperature. The heat flux on the surface of drilling tools were predicted and estimated. The numerical calculation and experimental results show the reliability of the inverse method. Piotr [28] presents a very practical method for solving inverse transient heat flow problems. The finite element software (ANSYS) (ANSYS Company, Pennsylvania, PA, USA) is used to reconstruct the transient temperature field based on the inverse calculation of the measured temperature points. Wang [29], on the basis of the two-dimensional heat transfer theory, established a heat conduction model of spherical bone abrasive tools. The distribution of heat flux was predicted by inverse calculation of experimental data and the time-varying characteristics of heat flux were considered. In a word, various inversion methods have become useful tools for the study of heat transfer processes in the fields of science and engineering.

At present, most scholars use sensors for signal processing to diagnose machine faults [30,31,32,33]. The on-line diagnosis of preload classification of hollow ball screw on the basis of sensor signals has also been studied by scholars [34]. Therefore, previous studies provide a good idea for this paper. The heat source load and temperature field of mechanical system can be estimated by the on-line measurement of temperature sensors. Furthermore, most of the thermal analysis research is focused on the spindle, and the real-time change of the position of the moving nut (moving heat source) for the ball screw feed system makes it difficult to identify the heat source. According to previous scholars’ research, this paper establishes a method for estimating the heat source of the ball screw feed system under an unsteady thermal effect by combining the data measured by temperature sensors and the dynamic thermal network model. Using the experimental data of the surface measuring points temperature and the position data of the moving nut, the dynamic change of the heat source and temperature field of the feed system under actual working conditions is simulated and estimated. At the same time, the finite element model of the feed system was established, and the heat generation of heat sources were optimized by using ANSYS parametric design language (APDL) based on the experiment data. Finally, the two estimation methods were validated by comparison with the experimental data [6,7,8,9].

## 2. Experimental Process

**Figure 1:**
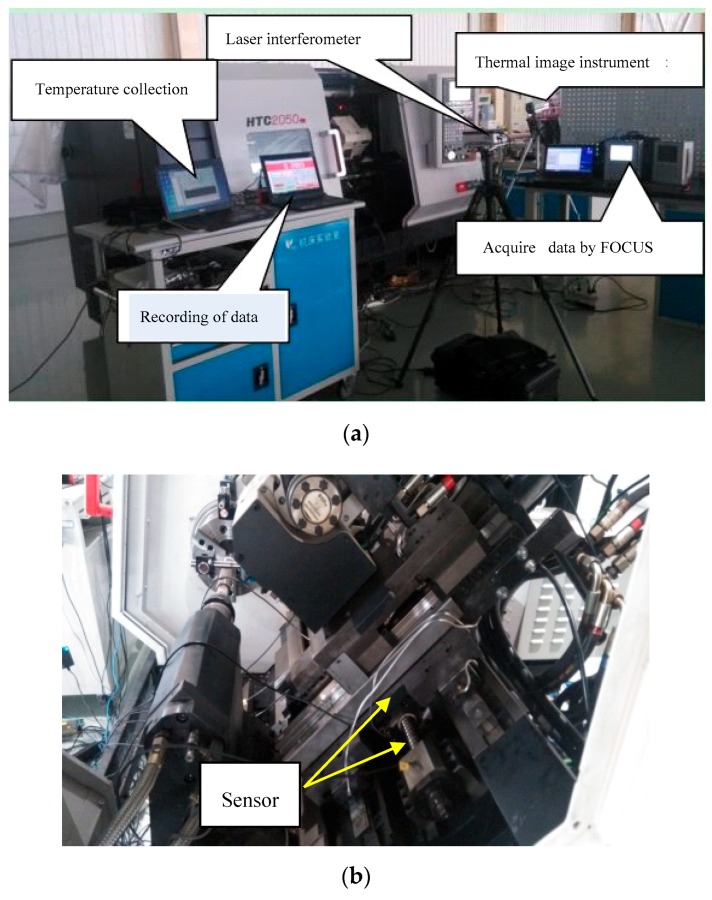
Experimental setup for measuring thermal characteristics. (**a**) Experimental equipment; (**b**) thermal sensor location.

In order to study and evaluate the heating characteristics of the ball screw feed system in working state, the experimental device is shown in Figure 1. The test object is the z-axis ball screw feed system of HTC2050i NC lathe, and the numerical control system is FANUC 0I Mate-TD, with the maximum feed speed of 2400 rpm. The bearings are special bearings for the ball screw of NSK manufacturing enterprise.

**Figure 2:**
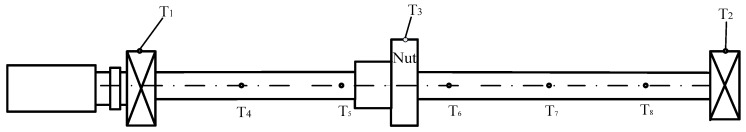
Distribution of measurement points.

The initial material properties of components are presented in Table 1 [13,22]. To analyze the heating characteristics of the heat source for the feed system, the experimental temperature points of the system should be reasonably selected, and the temperature sensors should be located as close as possible to the heat source to accurately reflect the temperature changes of each heat source. The experiment measuring points were placed as shown in Figure 2. Three T-type magnetic adsorption thermocouples are used to detect the temperature, and the thermocouples uncertainty is ±0.5 °C. The thermocouple sensors were used to detect the surface temperature T1 on the rear end bearing and the front end bearing temperature T2, as well as the temperature measuring point T3 on the screw nut. Furthermore, the temperature obtained from the thermocouple was collected and sent back to the computer for processing results. To measure the temperature of the ball screw shaft, an infrared thermal with high precision and fast response characteristics was used. The FLUKE thermal imaging instrument type is Ti110, the uncertainty of IR camera measurements is ±2%, and the emissivity is set to 0.95. In order to obtain the temperature data on the screw shaft, the temperature points T4–T8 on the surface of the lead shaft were picked up by thermal imager with isometric distance (the distance between the points on the rotating shaft is 100 mm). At the same time, PC104 computer were connected with CNC through network cable interface. FOCAS function interface program runs on PC104 industrial control, realizing the position of the recording nut in the working process. During the test, the feed system has no machining process. The feed system was tested in 3600 s at three speeds (500, 1000 and 1500 rpm) to record the position signal and temperature data signal.

## 3. Dynamic Thermal Network Modeling

### 3.1. Arrangement and Modeling of Thermal Nodes

**Figure 3:**
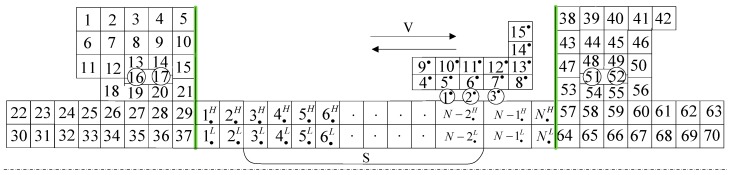
Thermal network arrangement of the ball screw system.

The heat transfer of feed system components is very complicated. According to the characteristics and the principle of symmetry of the ball screw feed system structure, the typical temperature node was selected to establish its thermal network. Thermal contact resistance is the resistance that hinders heat exchange between nodes. On the basis of the structure and material parameters of the feed system, the thermal resistance value was determined and calculated, and the transient temperature field was solved by establishing the equation. The thermal network of temperature nodes in the feed system of CNC machine tools is established in this paper. The arrangement of temperature nodes in the feed system was shown in Figure 3. There is a small part of heat flowing into the machine tool worktable and so on. In establishing the model, we assume that a small part of the heat entering the machine body and the worktable was ignored.

Since the whole system was divided into fixed heat source and moving heat source, the fixed heat source is the bearing ball, and the moving heat source is the ball screw nut. Therefore, the whole dynamic thermal network model was divided into three parts: the bearing end node, the moving node and axis middle segment node. The middle node of the screw shaft was divided into upper and lower parts. The contact between the upper part and the moving node of the screw nut should change the contact position dynamically, and the contact node needs to be updated and changed constantly when establishing the equation, so the solution is a dynamic equation. The movement position of the screw nut was updated in real-time based on the data captured by the experiment in the Section 2.

**Figure 4:**
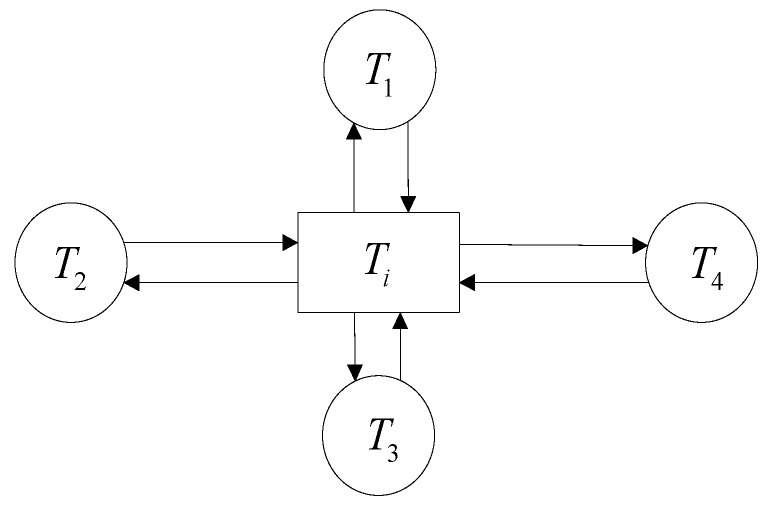
The temperature of a node heat balance system.

Each node satisfies the heat balance equation, as shown in Figure 4. The finite-difference (FD) equation of each node is obtained by applying the energy conservation equation for the node region [35].

(1) $$Q_{i}-C_{i}\rho_{i}V_{i}\frac{dT_{i}}{dt}=\sum_{ij=1}^{n}\frac{\left(T_{i}-T_{ij}\right)}{R_{i-ij}}\;i=1,2,\cdots ,m.$$

Here, $Q_{i}$ is the heat generation rate of the node heat source; $C_{i}$ is the specific heat capacity of the material; $\rho_{i}$ is the density of the material; V is the volume of the node related material. $T_{i}$ is the temperature of node i, and $T_{ij}$ is the node temperature in contact with node i; $R_{i-ij}$ is the thermal resistance coefficient between nodes i and *j*th, respectively. The heat balance differential equations of all nodes in the feed system were established. Using the Rung–Kutta method, MTALAB 2014 software (Business mathematics software produced by American MathWorks Company, Massachusetts, MA, USA) was programmed to solve the established differential equation. Here, the distribution relation of heat generation rate for the ball heat source was discussed according to Burton and Staph [36], 50% of the heat caused by friction is transferred to the balls, while the other 50% is transferred to the ball contact part.

### 3.2. Heat Transfer Model

Heat conduction, heat convection, and radiation are the main forms of heat transfer. Thermal radiation has little effect on the heat transfer of the feed system, so the influence of thermal radiation can be ignored. In the working process, the thermal conduction between bearing housing, the inner and outer rings of the bearing, the bearing balls, and the lead screw need to establish thermal resistance coefficient as follows:

1. The thermal resistance of the bearing outer ring, inner ring, and bearing housing of the bearing installed on the feed shaft is calculated as follows:
  (2) $$\left\{ \begin{array}{ll} (\ln(\frac{d_{I}}{d_{o}}))/2\pi k_{I}L & \mathrm{Radical}\;\mathrm{condunction}\\ \Delta L/Ak_{I} & \mathrm{Axial}\;\mathrm{condunction}\; \end{array}\right.$$
  in which $d_{I}$, $d_{o}$ are inner radius and external radius of cylindrical body, $k_{I}$ is the thermal conductivity and *L* is the natural length. ΔL and A stand the length of heat exchange and surface area.
2. The thermal contact resistance $R_{b}$ is related to the ball parameters and preload and working temperature. Thermal contact resistance between the balls and outer/inner ring can be defined [37]. At the same time, the thermal resistance between the ball and raceway of screw nut is also calculated as follows:
  (3) $$\left\{ \begin{array}{l} R_{b}=\frac{1}{2\pi ak_{b}}K(e,\frac{\pi }{2})+\frac{1}{2\pi ak_{r}}K(e,\frac{\pi }{2})\\ K(e,\frac{\pi }{2})=\int_{0}^{\frac{\pi }{2}}\frac{d\theta }{\sqrt{\left(1-(1-\frac{a^{2}}{b^{2}})\sin^{2}\theta \right)}} \end{array}\right.$$
  where $k_{b}$, $k_{r}$ are the thermal conductivity of ball and ring, a, b are the long/short axis of contact ellipse formed.
3. The assembly relationship between the shaft and bearing bore is interference fit, and the thermal resistance of the contact between the inner ring and shaft is
  (4) $$R_{h}=\frac{1}{Ah_{c}}=\frac{L_{g}(k_{d1}+k_{d2})}{2A\cdot A_{R}^{*}k_{d2}k_{d1}}.$$
  Here, A is the apparent contact area, $A_{R}^{*}$ is the real contact between two contact surfaces. $L_{g}$ is the void thickness of two contact surfaces. $k_{d1}$ and $k_{d2}$ are the thermal conductivities of the materials of the inner ring and the shaft.

The thermal conductivity of the joint surface contact is related to temperature, and the contact thermal resistance is:

(5) $$(\begin{array}{l} R_{\mathrm{co}}=\frac{1}{Ah_{\mathrm{co}}}\\ h_{\mathrm{co}}=\frac{1}{\frac{g_{r}}{h_{r}}+\frac{g_{o}}{h_{a}}} \end{array}$$

where $g_{r}$, $g_{o}$ are the thickness between bearing outer ring and circumferential air $h_{r}$ and $h_{a}$ are heat transfer coefficient [38]. The heat transfer coefficient is defined as:

(6) $$h=Nuk_{a}/d$$

where d is the equivalent diameter, and Nu is the average Nusselt number. However, the formula of Nu for different contact surfaces is dissimilar. $k_{a}$ is the thermal conductivities of the materials.

For the surface of the shell of the feed system, the heat is exchanged with the surrounding air in the form of a natural convection heat transfer, which is the same as the natural convection in the vertical plane. Then, the equation established in Reference [39] is determined.

(7) $$Nu=\left\{\frac{0.387{\left(Ra\right)}^{1/6}}{{\left[1+{\left(0.492/\mathrm{Pr}\right)}^{9/16}\right]}^{8/27}}+0.825\right\}$$

where Ra is the Rayleigh number and Pr is the Prandtl number.

Natural convection heat transfer occurs between the outer surface of the bearing seat and the surrounding air to exchange heat and emit heat. Usually, we regard the bearing housing as a horizontal cylinder, but due to the asymmetry of the air flow, the local heat transfer coefficient changes along the circumference direction. The coefficients are expressed as follows [40]

(8) $$Nu=\left\{\frac{0.387{\left(Ra\right)}^{1/6}}{{\left[1+{\left(0.559/\mathrm{Pr}\right)}^{9/16}\right]}^{8/27}}+0.6\right\}.$$

The forced convection coefficient for the rotating shaft can be estimated by Becker’s equation [41]

(9) Nu=0.6366 (RePr)1/2.

## 4. Inverse Method Solution of Heat Source by Genetic Optimization (GA)

According to Equation (1), the heat equilibrium differential equation of each node can be established to solve the entire differential equations, but the nonlinear dynamic change of heat source is not a fixed value under working conditions. Therefore, the inverse problem method is used to estimate the heat generation rate of the feed system based on the temperature data measured near the surface of the heat source. The simulation results (node 3, 40, 15 * on the thermal network) are compared with the temperature (T1, T2, T3) measured by the sensor experimental points, the simulated results points corresponding to the experiment are extracted one by one. The genetic optimization algorithm gradually circles to approximate the real heat source of heat generation rate until it meets the accuracy requirements and outputs the simulation optimization results.

Unknown three heat sources, heat sources generated at front and rear bearings, and the screw ball for a period time, are regarded as:

(10) $$Q={\left[q_{1}\;q_{2}\;q_{3}\right]}_{j}\;t_{j}\leq t\leq t_{j+1}.$$

When the objective function is minimized relative to each unknown parameter, the solution of the inverse problem can be obtained. For the moving heat source, the load position must be updated in real time according to the position changes measured in Section 2.

The objective function is defined to solve the inverse problem. The optimized objective function is as follows:

(11) $$\begin{array}{l} \min\;F(x)=\sum_{i}\sum_{j}{\left(T_{ij}^{EM}-T_{ij-k}^{GA}\right)}^{2}\\ s.t.\;q_{m}^{\min}\leq q_{m}\leq q_{m}^{\max}\;m=1,\;2,\;3 \end{array}.$$

In Equation (11), $T_{ij}$ is the temperature of the given point *i* of the *j*th sampling time step; the superscripts EM and GA respectively represent the experimental measurement results and the simulation results of genetic algorithm, and the lower and upper bounds of the heat source heat generation rate are $q_{m}^{\min}$ and $q_{m}^{\max}$, respectively.

The realization of genetic algorithm obtains any initial population according to the set parameter coding. Each individual in the population corresponds to a fitness value, and then simulates the survival of the fittest in nature through a series of genetic operations such as selection, crossover and mutation, and finally finds the optimal result.

**Figure 5:**
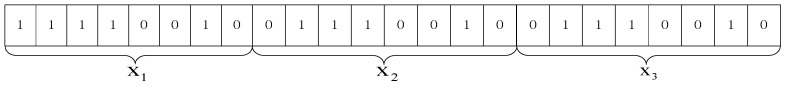
Binary representation in Genetic Optimization (GA).

1. Binary coding: The design variables defined by optimization problems are coded as the values of the design variables. In this paper, a finite length binary string is used to represent the parameters of each solution. So a sufficiently long chromosome can outline all the features of the design variable as shown in Figure 5.
  From the binary string to the real number mapping, the binary string number must be calculated first. To achieve the desired accuracy, the binary strings can be determined by the following formula:
  (12) $$2^{n}\leq \left(x_{m}^{\max}-x_{m}^{\min}\right)\times r\leq 2^{n+1}.$$
  Binary string to real number can be decoded according to Formula (12):
  (13) $$x_{i}=x_{m}^{\min}+\frac{x_{m}^{\max}-x_{m}^{\min}}{2^{n-1}-1}\sum_{j=1}^{n}\left(a_{i}\cdot 2^{j-1}\right)$$
  where $a_{i}\in \left[0,1\right]$.
2. Generate initial population: In order to satisfy the constraints and the diversity of the population, the initial solution of *K* groups is randomly generated as the size parameter of the population. These *K* individuals are the initial points of iteration, and the results satisfy the precision to terminate the iteration evolution process.
3. Fitness Arrangement: The objective function to be solved is directly transformed into fitness function correlation method. According to the size of K group objective function, the individual objective function is sorted from small to large.

The genetic operation of optimization process must go through crossover, mutation, and selection.

**Figure 6:**
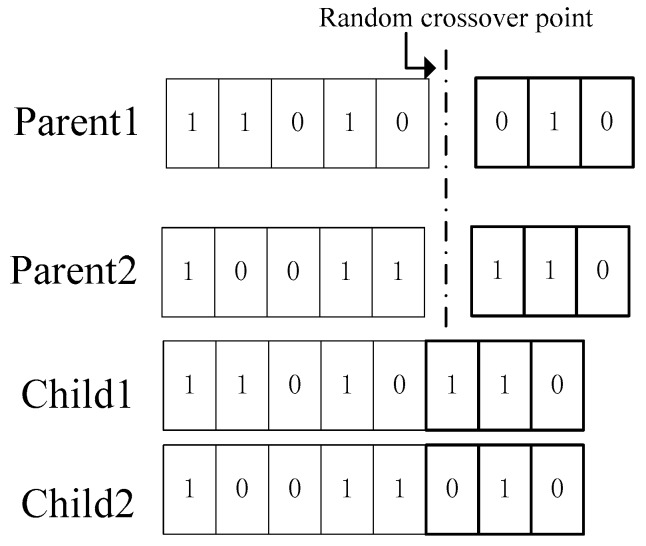
Schematic diagram of crossover.

Crossover is to choose two individuals for information exchange. Cross-selection of parental gene chromosomes produces new offspring. As shown in Figure 6, the two chromosomes were randomly selected from the K group by crossover.

**Figure 7:**
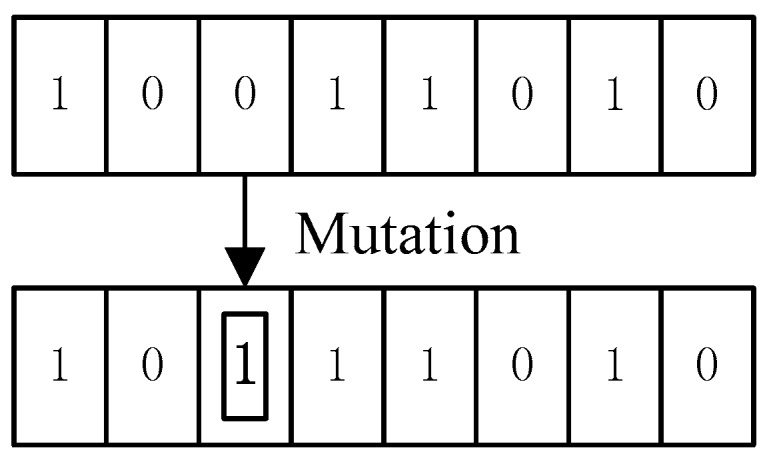
Schematic diagram of mutation.

Mutation is a random modification of binary strings, and the mutation operation reverses the gene value at certain loci, i.e., 0–1 or 1–0, as shown in the Figure 7. The mutation operation ensures the diversity of the population and accelerates the convergence rate of the optimal solution.

The ranking method is chosen by ranking the fitness of each individual. Each chromosome randomly generates numbers within a range. Each chromosome randomly generates numbers in a range of [0,1], and if it is less than the ranking probability, it will be eliminated, otherwise the binary coding of the chromosome will be retained. Therefore, the higher the ranking, the less likely it is to be eliminated. The formulas are as follows:

(14) $$p_{i}=\frac{R_{i}}{k}$$

where $p_{i}$ is the probability that the *i*th individual will be eliminated; $R_{i}$ is the fitness ranking of the individual.

**Figure 8:**
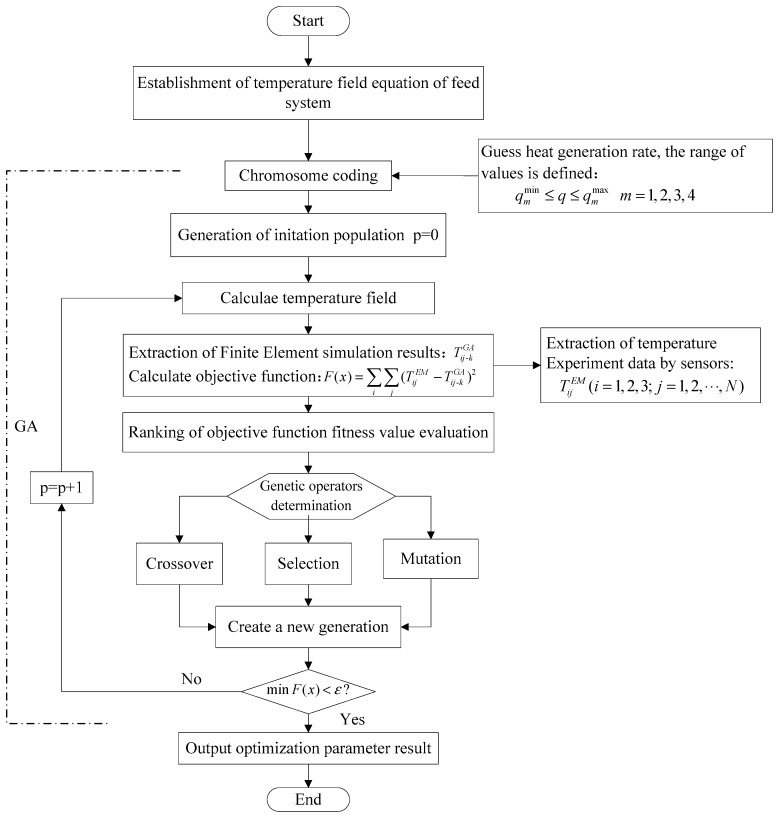
Flow chart of dynamic thermal network combined with GA optimization.

The optimization process is realized by MATLAB programming. The flow chart of the realization of the heat generation rate of the whole genetic algorithm is shown in Figure 8.

## 5. Simulation Results and Verification

### 5.1. Simulated Identification by FEM Combined with Genetic Algorithms (GA)

**Figure 9:**
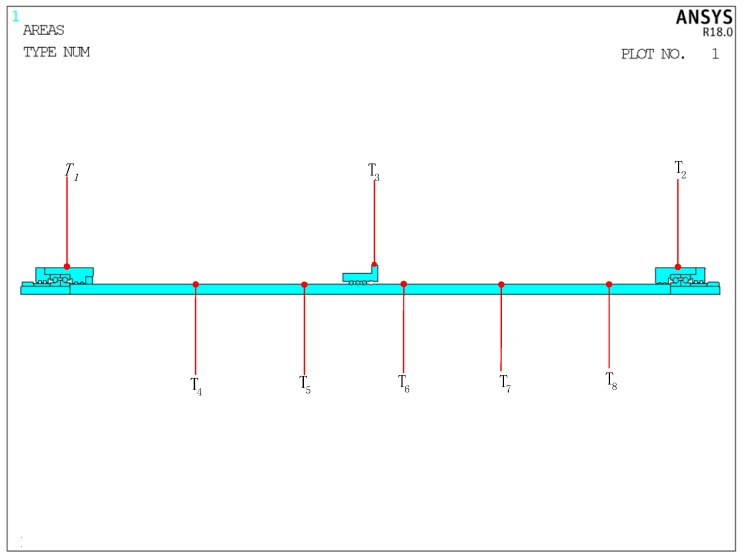
Location points T1–T8 in ANSYS software.

In order to verify the feasibility of the dynamic thermal network modeling method for identifying heat generation rate, this paper also applies the Section 4 genetic algorithm to optimize identification using finite element software (ANSYS) and compares these two numerical calculation methods. The ball screw feed system consists of multiple parts assembly, and the moving load under actual working conditions increases the complexity of the heat source estimation in the temperature field. Because of the principle of structural symmetry and the shortening of the calculation time, this paper establishes a two-dimensional model of the ball screw feed system as shown in the Figure 9. The ANSYS parametric design language (APDL) is used to program the model to apply boundary conditions, loads, and so on. The basic idea is as follows: The nuts’ stoke are divided equally in a space scale, and the position of the thermal load is updated by testing the position change of the nut recorded by experiment. When the heat load is used in a certain section, the heat generation rate load is applied at that location and the convective heat transfer load is applied at other locations. When moving to the next position, the last calculated calculation data is imported, the heat generation rate load of the previous section is deleted and the convective heat transfer load is applied, and the heat generation rate load is applied at this location. This is repeated until the end of the stroke is reached, then reversed to achieve the loading of the moving heat load.

Based on the energy conservation law of the first law of thermodynamics, the governing equations of the two-dimensional transient heat conduction are as follows [42]:

(15) $$\frac{\partial }{\partial y}\left(k_{y}\frac{\partial T\left(x,y,t\right)}{\partial y}\right)+\frac{\partial }{\partial x}\left(k_{x}\frac{\partial T\left(x,y,t\right)}{\partial x}\right)+q_{1}+q_{2}+q_{3}=\rho c\frac{\partial T\left(x,y,t\right)}{\partial t}.$$

The initial condition is:

(16) $$T\left(x,y,t\right)=T_{f}$$

and the boundary conditions are

(17) $$T\left(x,y,t\right)=T_{f}\;\mathrm{on}\;\mathrm{insulated}\;\mathrm{surfaces}$$

(18) $$-k\frac{\partial T\left(x,y,t\right)}{\partial x}=h\left(T\left(x,y,t\right)-T_{f}\right)\;\mathrm{on}\;\mathrm{the}\;\mathrm{convective}\;\mathrm{surface}\;\mathrm{everywhere}.$$

Here, T(x,y,t) indicates the domain for the ball screw feed system. $T_{f}$ is the surrounding temperature. *k* is the thermal conductivity of the material, and ρ and c are the density and heat capacity of the material, respectively. $q_{1}(t)$, $q_{2}(t)$ and $q_{3}(t)$ give the heat generation at the front and rear bearings and screw ball. h is the convection coefficient. More details for calculating h are presented in Section 3.2.

**Figure 10:**
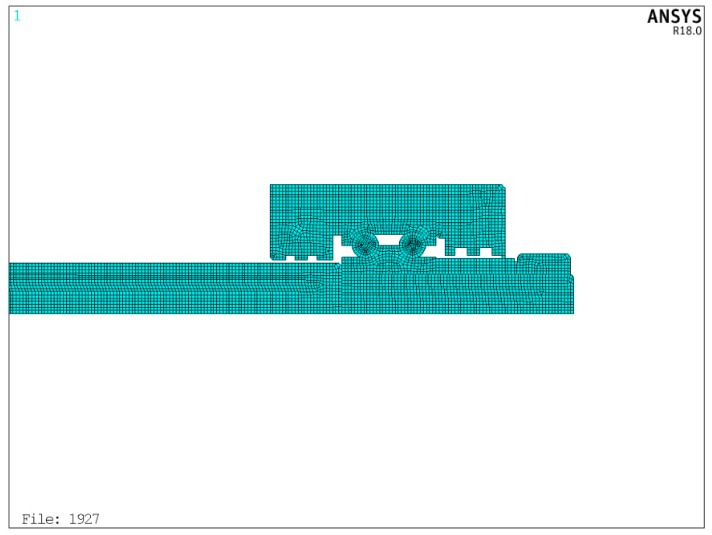
Mesh size of feed system.

Figure 9 depicts the finite element mesh model of the feed system. It can be seen that the temperature of the feed system has three points on the outer surface and five points on the feed axis (P1–P8), which are consistent with the experiment position (T1–T8). Because the mesh size affects the calculation time and accuracy in ANSYS, the mesh size should be reasonably selected before solving the temperature field. The number of grids should be increased reasonably at the contact position of heat source. Figure 10 shows the partially meshed feed system.

The three unknown heat sources generated for a period time are regarded as:

(19) $$Q={\left[q_{1}^{FEM}q_{2}^{FEM}q_{3}^{FEM}\right]}_{j}\;t_{j}\leq t\leq t_{j+1}.$$

Because the mesh size affects the calculation time and accuracy in ANSYS, the mesh size should be reasonably selected before solving the temperature field. Figure 10 shows the meshing of the bearing end. Then, according to the optimization method of Section 4, the FEM simulation results are extracted each time and compared with the temperature of the experiment points measured by the sensors. The optimal cycle finds the heat generation rate of heat source which is closest to the actual working condition. In the ANSYS software, the apdl command stream language is used to establish a genetic algorithm based optimization search algorithm.

### 5.2. Identification and Verification of Inverse Results

**Figure 11:**
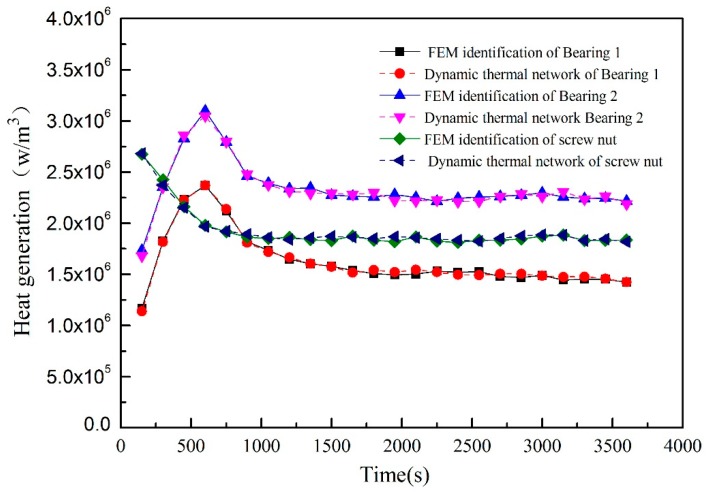
Heat generation of the heat sources identified by different calculation method.

**Figure 12:**
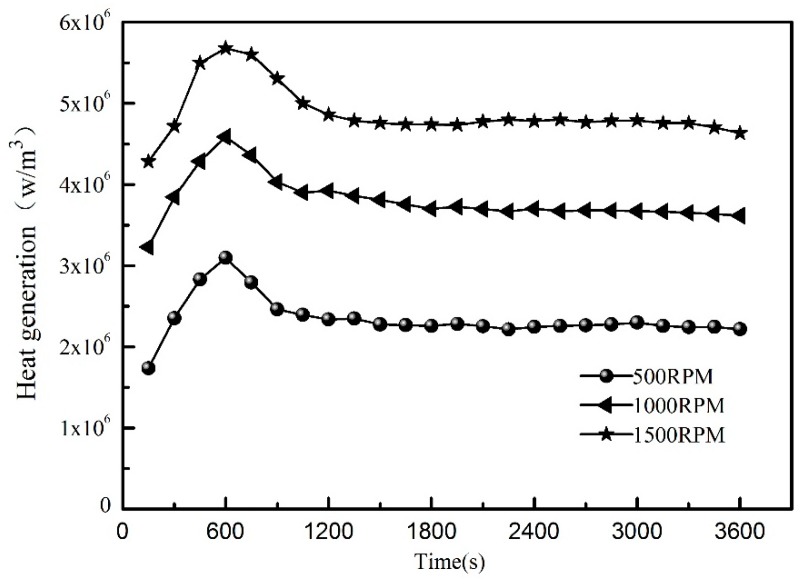
Estimated heat sources of front bearing at different speed.

Based on the estimation model described in this paper, Figure 11 shows the dynamic thermal network method and the finite element software of two numerical calculation methods to optimize the heat generation of the identification results at the feed rate of 5 m/min. Among them, the solid line is the FEM identification result, and the dotted line is the dynamic thermal network identification result. The two kinds of calculation results are quite consistent. The friction heat generation rate increases rapidly from zero to the maximum, and then the friction heat generation rate continues to decline until it reaches the steady-state value. According to the trend, it can be easily inferred that the preload causes the frictional heat generation of the rolling bearing and screw nut under working conditions to increase significantly, and the heat generation and heat dissipation rate are unbalanced. As the preload and kinematic viscosity slowly decrease, the heat generation rate decreases, and the internal stability of the bearing is gradually achieved. However, the FEM method takes more time to optimize and the dynamic thermal network method takes less time. As illustrated in Figure 12, the predicted heat generation of the front bearing at different feed speeds is not a fixed value. Hence, the traditional calculation method is not very accurate to predict the temperature field. The inverse problem solving method provides a good idea.

**Figure 13:**
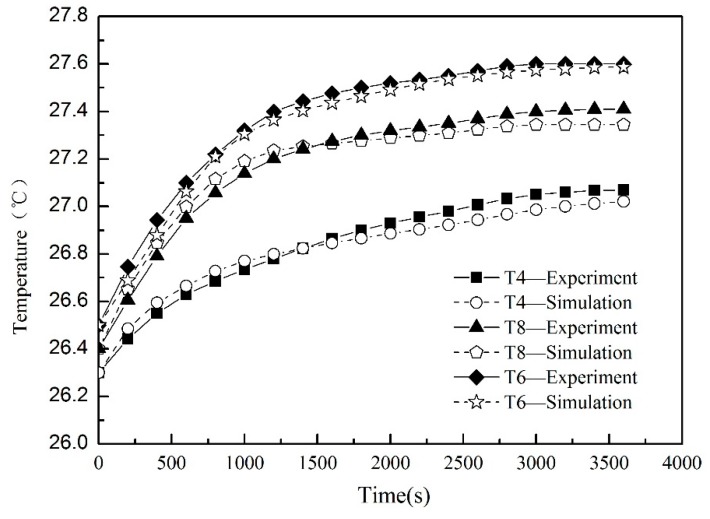
Comparison of simulated and experimental temperature at verified points.

Before the simulation, the experiment was carried out as described in Section 2, and the thermal imager measured the temperature distribution. Other temperature points on the axis (T4–T8) can be used to verify the simulation results and verify the accuracy of identification results for the heat generation rate and temperature field of the system. Figure 13 is a comparison of estimated and measured temperature points at the feed rate of 5 m/min. It can be seen that the temperature estimated by the dynamic thermal network agrees well with the experimentally measured temperature data. Initially, the temperature rises rapidly and then becomes constant, showing a fairly close consistency. At the same time, it can be seen that when the heat generated by the heat source and the heat dissipated reach equilibrium, the temperature approaches the steady state. As time goes on, more frictional heat dissipates throughout the bearing housing and shaft. The identification accuracy of the temperature field verifies the feasibility of this inverse problem solving method.

## 6. Conclusions

This paper presents a method for estimating the heat source of the feed system of CNC machine tools under actual working conditions. The inverse calculation method combining dynamic thermal grid network with genetic algorithm is used to predict the time-varying heat source by the on-line monitoring of the temperature of the heat source by temperature sensor. The inverse calculation and estimation analysis of the heat source and temperature field distribution of the feed system are realized. Comparing this method with the FEM prediction calculation method, it shows good consistency. Both methods can evaluate the heat source, but the FEM calculation is relatively complex and time-consuming. The predicted temperature on the screw shaft is compared with the temperature measured by the experiment, showing a significant consistency. The results show that the method is suitable for analyzing the heat source and temperature distribution of the high-speed feed system at different rotational speeds and has good reliability. Therefore, the temperature field can be accurately estimated and extend to control the excessive temperature of the bearing and compensate the thermal error of the feed shaft. In this paper, a simple and convenient method is proposed to estimate the thermodynamic behavior of ball screw feed system of machine tools. It provides a new idea for identifying the heat generation rate of the heat source under actual working conditions, and has a strong application value in practical engineering applications.

## Figures and Tables

**Table 1:** Material properties on parts.

Part	Density $\rho {\;\mathrm{kg}/m}^{3}$	Elastic Modulu E Gpa	Poisson’s Ratio v	Heat Capacity C J/(kg·°C)	Thermal Conductivity $k_{d}\;W/(m\cdot {}^{\circ}C)$
Screw nut	7810	2.07	0.3	553	50
Housing	7810	2.20	0.3	494	40
Ball	3200	3.20	0.26	800	11.63
Outer/inner ring	7810	2.07	0.3	450	40.1
Shaft	7860	2.12	0.298	485	42.3
Cage	7830	2.06	0.254	460	44
